# PPE38 Protein of *Mycobacterium tuberculosis* Inhibits Macrophage MHC Class I Expression and Dampens CD8^+^ T Cell Responses

**DOI:** 10.3389/fcimb.2017.00068

**Published:** 2017-03-13

**Authors:** Lu Meng, Jingfeng Tong, Hui Wang, Chengwu Tao, Qinglan Wang, Chen Niu, Xiaoming Zhang, Qian Gao

**Affiliations:** ^1^Key laboratory of Medical Molecular Virology, Institute of Biomedical Sciences and Institute of Medical Microbiology, Shanghai Medical College, Fudan UniversityShanghai, China; ^2^The State Key Laboratory of Respiratory Disease for Allergy at Shenzhen University, School of Medicine, Shenzhen UniversityGuangdong, China; ^3^Key Laboratory of Molecular Virology and Immunology, Institute Pasteur of Shanghai, Chinese Academy of SciencesShanghai, China

**Keywords:** *Mycobacterium*, PPE38, MHC class I, CD8^+^ T cells, macrophages

## Abstract

Suppression of CD8^+^ T cell activation is a critical mechanism used by *Mycobacterium tuberculosis* (MTB) to escape protective host immune responses. PPE38 belongs to the unique PPE family of MTB and in our previous study, PPE38 protein was speculated to participate in manipulating macrophage MHC class I pathway. To test this hypothesis, the function of mycobacterial PPE38 protein was assessed here using macrophage and mouse infection models. Decreased amount of MHC class I was observed on the surface of macrophages infected with PPE38-expressing mycobacteria. The transcript of genes encoding MHC class I was also inhibited by PPE38. After infection of C57BL/6 mice with *Mycobacterium smegmatis* expressing PPE38 (Msmeg-PPE38), decreased number of CD8^+^ T cells was found in spleen, liver, and lungs through immunohistochemical analysis, comparing to the control strain harboring empty vector (Msmeg-V). Consistently, flow cytometry assay showed that fewer effector/memory CD8^+^ T cells (CD44^high^CD62L^low^) were activated in spleen from Msmeg-PPE38 infected mice. Moreover, Msmeg-PPE38 confers a growth advantage over Msmeg-V in C57BL/6 mice, indicating an effect of PPE38 to favor mycobacterial persistence *in vivo*. Overall, this study shows a unique biological function of PPE38 protein to facilitate mycobacteria to escape host immunity, and provides hints for TB vaccine development.

## Introduction

Tuberculosis (TB) is an ancient disease caused by *Mycobacterium tuberculosis* (MTB), a pathogen that infects one-third of the world's population. Most infected people develop a long lasting protective immune response, while about 3–10% will develop active infections during their lifetime (Zumla et al., 2011).

Previous studies have established that CD4^+^ T cells and CD8^+^ T cells play key roles in controlling MTB infection (Lindestam Arlehamn et al., 2014; Jasenosky et al., 2015). CD4^+^ T cells, which recognize antigenic peptides derived from the phagosomal compartment in the context of major histocompatibility complex (MHC) class II molecules, are crucial to control of initial infection by MTB as well as preventing recurrence (Kaufmann et al., 2005). The higher mortality rates of HIV-MTB patients emphasize the essential immunity function of the class II MHC-restricted CD4^+^ T cells (Kaufmann et al., 2010; Bradshaw et al., 2016).

In addition to CD4^+^ T cells, CD8^+^ T cells are also essential in the host defense against MTB infection. Classically, CD8^+^ T cells recognize antigens derived from proteins in the cytosolic compartment through MHC class I. First, cytosolic antigens are digested proteolytically by the proteasome and other proteases. After binding with antigen peptide transporter (TAP) complexes, the peptides are then transported into the endoplasmic reticulum (ER). In the ER, the peptides form a complex with MHC class I and are exported to the plasma membrane, where they are recognized by CD8^+^ T cells (Heemels and Ploegh, 1995; Rock et al., 2002; Abele and Tampe, 2009). In animal infection models, mice lacking β2-microglobulin (β2 m), which is required for assembly of MHC class I heavy chain, died quickly after MTB infection (Flynn et al., 1992). Moreover, antigen processing (TAP)-1(−/−) or CD8 (−/−) knockout mice with disruptions of the MHC class I antigen processing pathway or/and functional CD8^+^ T cells are more susceptible to MTB infection (Flynn et al., 1992; Behar et al., 1999; Sousa et al., 2000; Turner et al., 2001; Urdahl et al., 2003). Moreover, both murine and human CD8^+^ T cells could be activated by mycobacterial antigens (Ags) (Stenger et al., 1997; Lalvani et al., 1998; Kamath et al., 2004, 2006; Irwin et al., 2005; Woodworth et al., 2011). In TB patients, CD8^+^ T cells were found in granulomas, indicating the recruitment of CD8^+^ T cells post-MTB infection (Munk and Emoto, 1995; Tully et al., 2005).

Upon phagocytosis by macrophages, MTB survives and proliferates in phagosomes. Although MTB Ag presentation by MHC class I is not the canonical mode, there is increasing evidence that endocytosed MTB Ags can be presented through MHC class I, a process known as cross-presentation (Rock and Shen, 2005; Jensen, 2007). Additionally, the MHC class I-dependent process can also be activated once MTB escapes or MTB proteins are exported from the phagosome into the cytosol (van der Wel et al., 2007; Behar et al., 2011). Furthermore, apoptotic vesicles generated from infected macrophage cells can be taken up by dendritic cells, where the peptides are shuttled into the MHC class I pathway (Schaible et al., 2003; Winau et al., 2006).

PPE38 protein belongs to the highly polymorphic proline-proline-glutamic acid (PPE) family, which consists of 69 PPE proteins encoded by the *M. tuberculosis* H37Rv (Cole et al., 1998). PPE proteins are considered as pivotal candidates for MTB vaccine development (Pajon et al., 2006; Bertholet et al., 2008; Lewinsohn et al., 2013), and at least 10 PPE proteins are able to elicit T cell responses (Sampson, 2011). Recently, a study described that PPE65 inhibits both CD4^+^ and CD8^+^ T cell activity by suppressing Th1 Cytokines (Khubaib et al., 2016). However, it remains unclear whether MTB modulates MHC class I expression, which, in turn, influences CD8^+^ T cells activation.

The current study was undertaken to characterize the function of a MTB virulence factor, PPE38, which may inhibit the MHC class I process during mycobacterial infection. After comparing the proteomic profiles of macrophages infected by an *M. marinum ppe38* mutant (Tn-MmPPE38) or the wild-type (WT Mm), the expressions of TAP1, TAP2, and TAP binding protein isoform 1 (TAPBP) which are involved in MHC class I pathway, were significantly downregulated (Wang et al., 2013). Besides, macrophages infected with MTB or Bacille Calmette-Guerin (BCG) will present Ags through the TAP-dependent MHC class I presentation pathway (Teitelbaum et al., 1999), thus we speculated that the PPE38 protein may be involved in antigen presentation, which ultimately influences CD8^+^ T cells responses to escape host elimination. To test this hypothesis, we first investigated the expression of MHC class I in infected macrophages. Then, a mouse-*Mycobacterium smegmatis* infection model was used to measure the activation of CD8^+^ T cells in the spleen. In addition, we assessed the amounts of CD8^+^ T cells in infected organs and the *in vivo* survival of the bacteria. Overall, this study contributed to our understanding of TB pathogenesis, in particular how the bacteria escape the host CD8^+^ T cell-dependent immunity.

## Materials and methods

### Mice and cell lines

C57BL/6 mice and BALB/c mice were purchased from the Animal Center of Slaccas (Shanghai, China) and housed under specific pathogen-free conditions in the Animal Center of Fudan University. The experimental procedures followed the Guidelines for the Care and Use of Laboratory Animals from the National Institutes of Health and were approved by the Animal Care and Use Ethical Committee of Fudan University. All the procedures were carried out in accordance with the guidelines. The RAW264.7 cell line was purchased from the Cell Bank of the Chinese Academy of Sciences (Shanghai, China). Cells were cultured in Dulbecco's modified Eagle's medium (DMEM) (Gibco, Grand Island, NY, USA) supplemented with 10% fetal bovine serum (FBS), penicillin (100 U/ml) and streptomycin (100 mg/ml) and maintained at 37°C in a humidified incubator (5% CO_2_).

### Bacterial strains

*M. marinum* M strain (ATCC BAA-535) was used in our research. Strain Tn-MmPPE38 was generated by transposon mutagenesis of *Mycobacterium marinum* (*M. marinum*). Both Tn-MmPPE38 and its complemented strain (Comp-MmPPE38) were constructed as described previously (Dong et al., 2012). For generation of recombinant *M. smegmatis* expressing PPE38, the *ppe38* gene was amplified using PCR based on the genomic DNA sequence of *M. tuberculosis* H37Rv with specific primers (forward primer, F-5′-CGGGATCCTGGAGGGGTTGCGATGATTTTTGGATTTTTC-3′ and reverse primer R-5′-GGACTAGTACCGCTTCGGTGCACTTCATTTC-3′). The PCR product were digested with *BamHI* and *SpeI*, and cloned into pSMT3 to generate pRvppe38. The recombinant plasmid was introduced into *M. smegmatis* (Msmeg-PPE38 and Msmeg-V) via electroporation. Msmeg-PPE68 was constructed in our previous study (unpublished data).

### Bacterial sample preparation

*M. marinum* strains (WT Mm, Tn-MmPPE38, and Comp-MmPPE38) and Msmeg-V and Msmeg-PPE38 cultures were grown in Middlebrook 7H9 medium supplemented with 10% OADC and 0.05% Tween 80 till OD600~0.6. Bacterial cell pellets were harvested and washed with phosphate buffer saline (PBS). The single-cell suspension of bacteria was prepared by subjecting cells to vortex 5 min with 1 mm glass beads, and passage through a 5-μm syringe filter.

### Cell culture

The murine macrophage cell line RAW264.7 (ATCC TIB71) was maintained at 37°C with 5% CO_2_ in Dulbecco's Modified Eagle's Medium (DMEM), supplemented with 10% fetal bovine serum (FBS) and 10 mM HEPES. Peritoneal macrophages (PMs) was isolated as described before (Lu et al., 2013). In brief, each mouse was injected with 1 ml of aged 3% thioglycollate medium via peritoneal injection. After 4–5 days, peritoneal cells were harvested and cultured in RPMI 1640 medium with 10% FBS at 37°C in 5% CO_2_.

### Infection of Raw264.7 macrophages by *Mycobacterium* strains

Prior to infection, RAW264.7 cells were seeded into 24-well plate at a density of 2 × 10^5^ cells per well and maintained at 37°C with 5% CO_2_. After overnight growth, the RAW264.7 cells were infected with prepared *Mycobacterium* strains. Spent media was replaced with 1 ml of fresh DMEM media containing 10% FBS and sufficient mycobacteria to achieve a multiplicity of infection (MOI) of 10 (i.e., 10 bacteria for one macrophage). The infection was allowed to proceed for 4 h for *M. marinum* at 32°C, or 2 h for *M. smegmatis* strains at 37°C in 5% CO_2_. The CFUs of mycobacteria strains used was enumerated by plating appropriate dilutions on Middlebrook 7H10 supplemented with 10% OADC (C7H10) agar plates.

### Flow cytometric analysis

Peritoneal macrophages (PMs) from C57BL/6 mice was isolated following published protocols (Bermudez et al., 1997; Overbergh et al., 2000). In brief, infected C57BL/6 mice were euthanized at 6 h post infection (hpi), 1 day post infection (dpi) or 3 dpi. PMs of each mouse were obtained by washing the peritoneal cavity with 5 ml cold PBS. Macrophages was collected in sterile 15 ml tubes and placed immediately on ice. RAW264.7 or PMs from infected C57BL/6 mice were harvested and washed with pre-chilled PBS, followed by centrifugation at 1,000 × g for 10 min at 4°C. The cells were treated with Fc Block (1:100) (BD Pharmingen, CA, USA) in PBS supplemented with 1% BSA and then incubated with Anti-Mouse CD11b FITC, F4/80, and Anti-Mouse MHC Class I (H-2Kb) PE (eBioscience, USA) on ice for 15 min in the dark room. For splenic CD8^+^ T cell analysis, at 6 dpi, spleens were collected and manually dissociated into single cell suspension. The staining procedure was performed as in the above, with CD44, CD62L. The cells were washed once using PBS+10% FBS and resuspended in 200 μl FACS staining buffer (eBioscience, USA), and analyzed using flow cytometers FACSCalibur (Becton Dickinson, USA). The data were analyzed using the Cell-Quest data analysis software (100,000 events per sample) and FlowJo.

### Real-time quantitative PCR

RAW264.7 RNA was purified using Trizol method. 1 μg RNA was converted to cDNA using PrimeScript™ II 1st Strand cDNA Synthesis Kit. PCR product was detected using SYBR Premix Ex Taq II Kit. Amplification cycle was applied as 95°C for 15 s, 55°C for 5 s and 72°C for 10 s. Primers sequences were as follows: GAPDH (forward primer: F-5′- AACGACCCCTTCATTGAC-3′ and reverse primer R-5′-TCCACGACATACTCAGCAC-3′). MHC class I (H-2K^b^) (forward primer: F-5′- TACCAGCAGTACGCCTACGAC -3′ and reverse primer R-5′- GCGTTCCCGTTCTTCAGGTAT-3′). qRT-PCRs were carried out by using a CFX96™ real-time PCR system (Bio-Rad) using the relative quantification methodology. The results were expressed as a ratio relative to the GAPDH level. All reagents were purchased from Takara, Japan.

### Mouse infection

Bacterial strains were dispersed in PBS and quantified with Colony forming units (CFU) counting. Six week old female C57BL/6 mice were infected with 2 × 10^7^ CFUs of either Msmeg-V or Msmeg-PPE38 via intraperitoneal injection according to previous report (Dheenadhayalan et al., 2006).

### Bacteria burden in organs

Bacterial loads in lungs, liver, and spleen were evaluated at 1, 6, and 9 dpi, respectively. Briefly, the organs were aseptically removed from euthanized mice and homogenized in sterile saline containing 0.05% Tween 80 (Sigma-Aldrich). Serial dilutions of homogenized organs were plated on C7H10 agar plates. Plates were incubated at 37°C and colonies were counted after 3 days.

### Immunohistochemical detection of CD8^+^ T cells in infected organs

The histopathology was carried out as described earlier (Higgins et al., 1999). In Brief, lungs, liver, and spleen of mice was aseptically removed from euthanized animals and were fixed in 2.5% glutaraldehyde-paraformaldehyde mixture. Sections were then stained with anti-CD8 (PE488) for visualization. DAPI nuclear staining was used to identify cells. Microphotographs were taken using an Olympus DP72 CCD camera (Olympus, Japan) attached to the microscope. A group of three mice were used at 6 dpi, and CD8^+^ T cells were counted manually from three different fields of each mice (×400 time magnified).

### Data statistics

All data were generated from three independent experiments. They were shown as Means ± SD and processed with Graphpad software. Student *t*-test (two tailed) was performed to test the significant differences between groups. *p* < 0.05 was considered as significant.

## Results

### Mycobacterium PPE38 protein reduced MHC class I expression in RAW264.7

**Figure 1:**
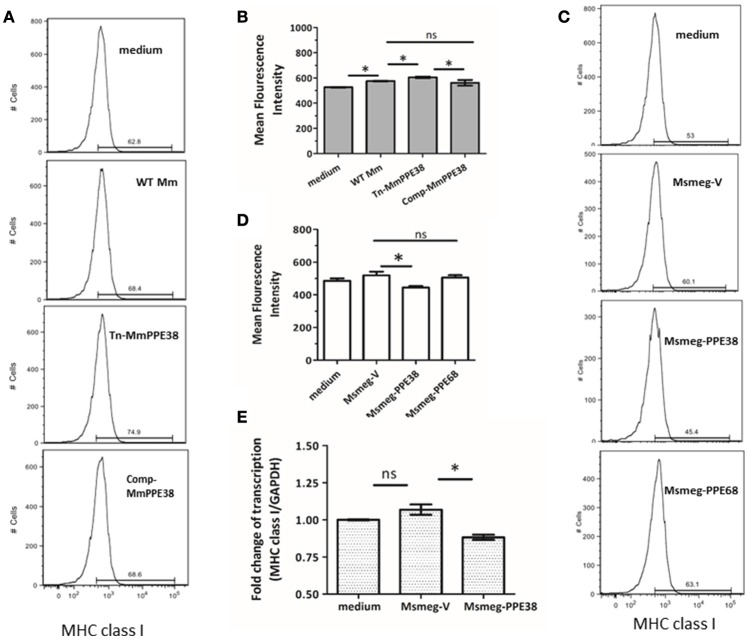
**PPE38 protein inhibited the MHC class I expression in macrophages**. RAW264.7 cells were infected with either *M. marinum* strains (WT Mm, Tn-MmPPE38, and Comp-MmPPE38, MOI = 1) or *M. smegmatis* strains (Msmeg-V, Msmeg-PPE38, and Msmeg-PPE68, MOI = 10). The expression of MHC class I was detected by FACS at **(A,B)** 4 hpi for *M. marinum* strains or **(C,D)** 2 hpi for *M. smegmatis* strains by FACS analysis. **(E)** Total RNA was isolated from infected RAW264.7 cells. The level of MHC class I was measured by qRT-PCR and expression levels were normalized to corresponding GAPDH levels. Data were generated from four independent experiments. ^*^p < 0.05.

Based on our previous data, which showed that the MHC class I pathway proteins TAP1 and TAP2 were significantly downregulated by PPE38 (Wang et al., 2013), we hypothesized that PPE38 might play a role in suppressing MHC class I expression. To verify this, RAW264.7 cells were infected with *M. marinum* (WT Mm), Mmppe38 mutant strain (Tn-MmPPE38) or the complemented strain (Comp-MmPPE38), respectively. As shown in Figures 1A,B, compared with the WT Mm, more MHC class I proteins were observed on the cell surface of macrophages after Tn-MmPPE38 infection, indicating that macrophage expression of MHC class I on the cell surface was inhibited by PPE38. Similar amounts of MHC class I protein to that of the WT Mm were observed when Comp-MmPPE38 was used. Furthermore, as *M. smegmatis* lacks an endogenous homolog of the MTB PPE38 protein, therefore, we expressed MTB PPE38 in *M. smegmati*s (Msmeg-PPE38) (Figures 1A,B) and then used it to infect macrophages. Similarly, the amount of MHC class I was significantly reduced in RAW264.7 cells infected with MS-PPE38, compared with the control strain harboring empty vector (Msmeg-V) (Figures 1C,D). We also detected the effects of another PPE protein, PPE68, on MHC class I expression, but did not observe similar effects. In addition to studies of cell surface expression of MHC class I, real-time quantitative PCR was used to measure MHC class I H-2K^b^ mRNA expression in macrophages after incubation with Msmeg-V or Msmeg-PPE38. As showed in Figure 1E, significantly reduced MHC class I transcript levels were observed when PPE38 was expressed, which was consistent with decreased MHC class I protein levels described above. Taken together, the results suggested that the PPE38 protein inhibited MHC class I synthesis in macrophages, most likely at the transcriptional level.

### MTB PPE38 protein inhibits MHC class I expression in peritoneal macrophages

**Figure 2:**
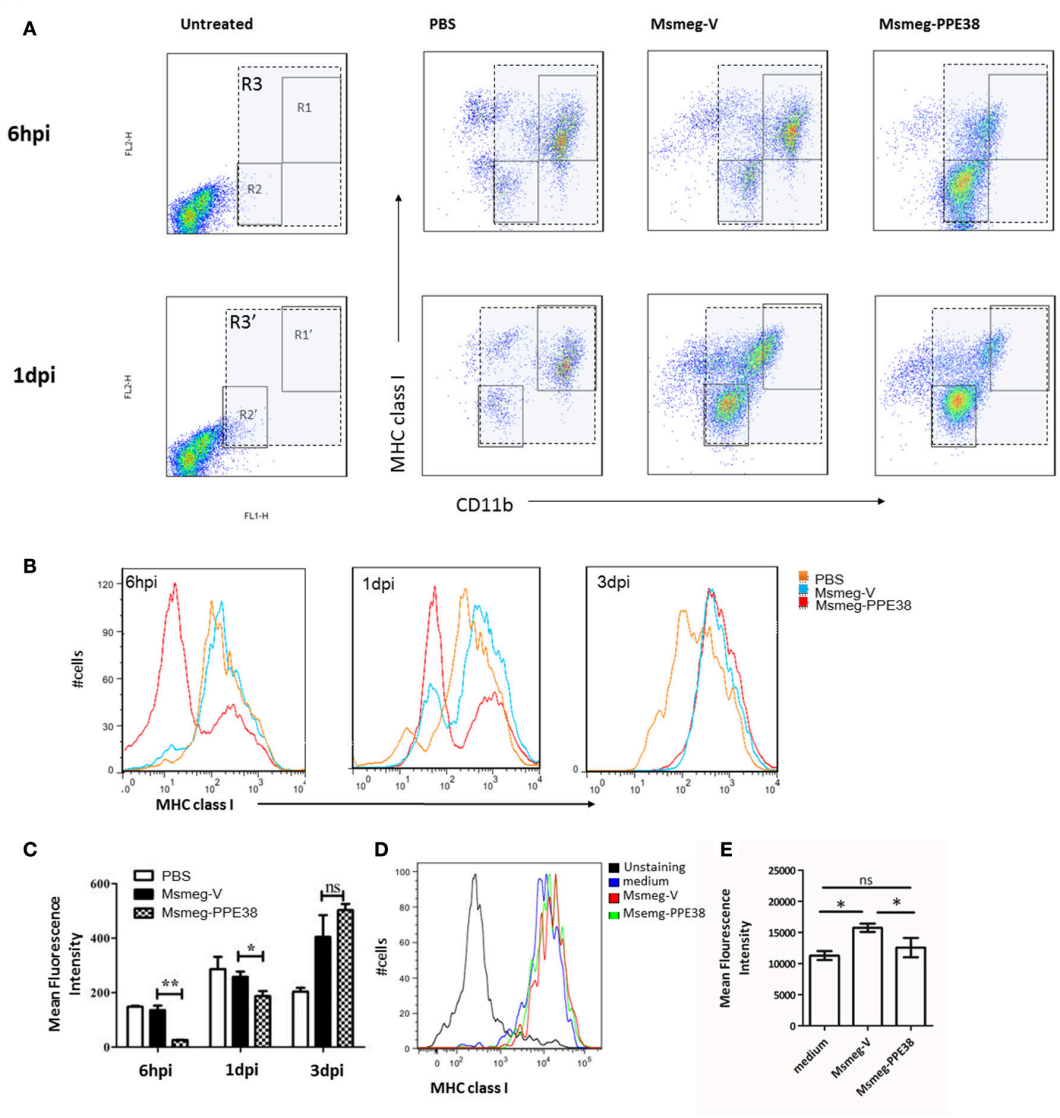
**PPE38 dampened the expression of MHC class I in peritoneal macrophages**. C57BL/6 mice were infected with Msmeg-V and Msmeg-PPE38 (2 × 10^7^), the peritoneal macrophages collected from infected mice were stained with FITC conjugated anti-CD11b mAbs, and PE-conjugated anti-MHC class I mAbs. **(A)** Expression of MHC class I were analyzed by FACS from 1^*^10^6^ cells at 6 hpi (gate R1–R3) and 1 dpi (gate R1′–R3′). Peritoneal macrophages were first gated as CD11b^+^ (gate R3 and R3′), then two subgroup of PMs were observed as CD11b^high^MHC class I^*high*^ (gate R1 and R1′) and CD11b^low^MHC class I^*low*^ (gate R2 and R2′). **(B)** Histograms and **(C)** bar graphs show the Mean Fluorescence Intensity of MHC class I of macrophages. **(D,E)** Isolated mouse peritoneal macrophages from mice pretreated with 3% thioglycollate medium were cultured for 24 h and then infected with Msmeg-V and Msmeg-PPE38 (MOI = 10). The amount of MHC class I was measured via FACS from 1^*^10^5^ cells. Bar graphs show the mean ± SD, representing three independent experiments. ^*^p < 0.05. ^**^p < 0.01.

Furthermore, the expression of MHC class I on mouse peritoneal macrophages (PMs) were also examined. After infection with Msmeg-V and Msmeg-PPE38, C57BL/6 mouse PMs were isolated and analyzed by fluorescence activated cell sorting (FACS). As shown in Figure 2A, we firstly gated CD11b^+^ cells comparing to the uninfected group. Then, the expression of MHC class I was evaluated, and two distinct groups (CD11b^low^ MHC class I^low^ and CD11b^high^ MHC class I^high^) PMs were observed. At 6 hpi, the frequency of CD11b^low^ MHC class I^low^ was significantly increased only in Msmeg-PPE38 group, indicating that PPE38 could play a role in the generation of CD11b^low^ PMs. Consequently, when PMs were gated as a single population based on CD11b and F4/80 expression (Figure 2), as shown in Figures 2B,C, the MHC class I level in PMs of the Msmg-PPE38 group was significantly lower than that in the PMs of the Msmeg-V group at 6 h post infection (hpi) and 1 day post infection (dpi). To assess the direct impact of PPE38 on MHC class I expression in PMs, isolated PMs from mice pretreated with 3% thioglycollate medium were cultured for 24 h, and then treated with Msmeg-V or Msmeg-PPE38. PMs infected with Msmeg-V significantly upregulated MHC class I, but PMs infected with Msmeg-PPE38 failed to do so (Figures 2D,E). Consistent with what we have observed in macrophage cell line (Figures 1D,E), we speculate that PPE38 could inhibit MHC class I expression in mycobacteria-infected macrophages.

### Decreased CD8^+^ T cells were elicited in C57BL/6 mice infected with *M. smegmatis* expressing PPE38 protein

**Figure 3:**
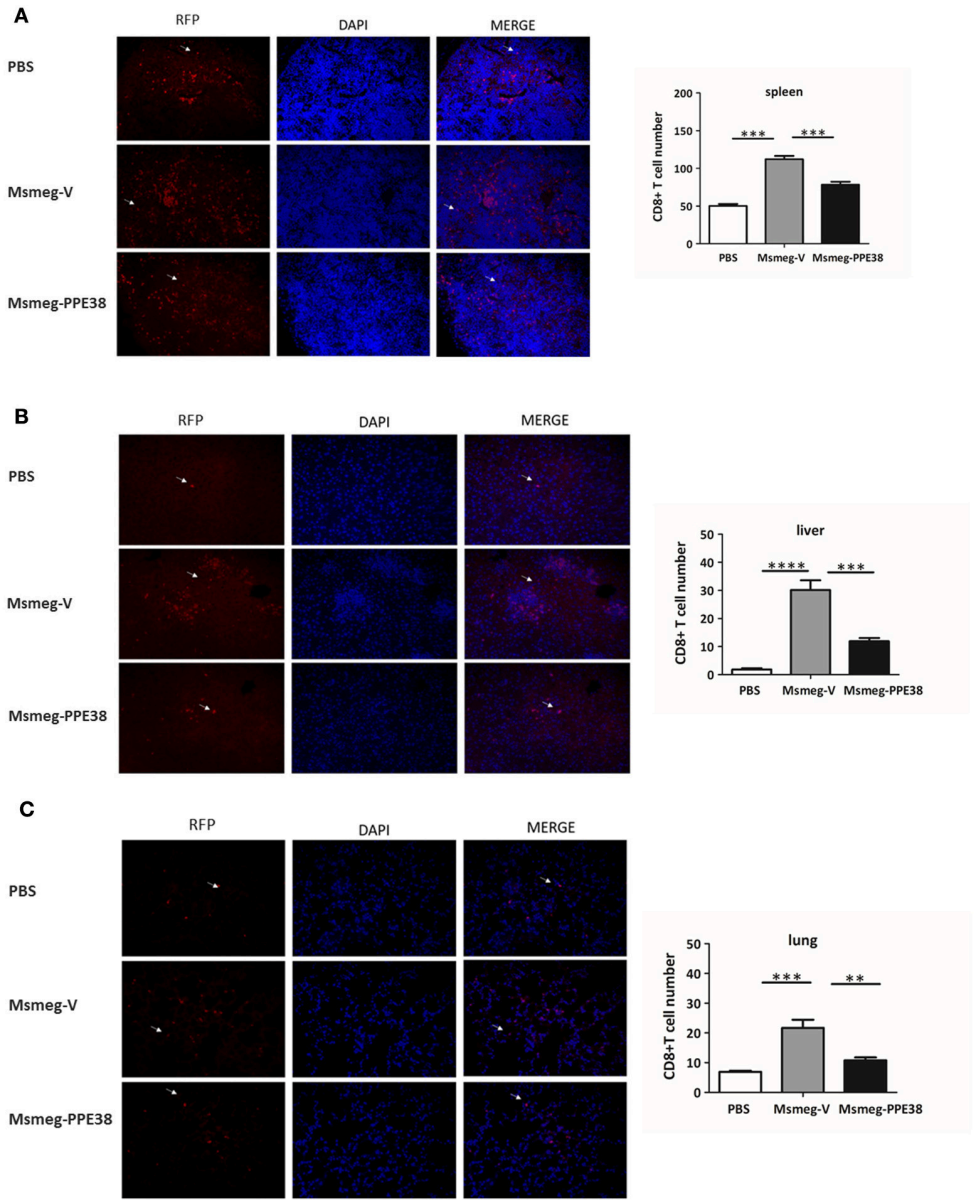
**C57BL/6 mice infected with Msmeg-PPE38 showed reduced distribution of CD8^**+**^ T cells in infected organs as compared to mice infected with Msmeg-V**. The **(A)** spleen, **(B)** liver and **(C)** lungs sections of C57BL/6 mice either treated with PBS or infected with 2 × 10^7^ of Msmeg-V or Msmeg-PPE38 at 6 dpi were stained with anti-CD8 antibody and DAPI. Histograms and bar graphs show the number of CD8^+^ T cells. Arrowheads show CD8^+^ T cell. Photographs of representative sections were shown (× 400 time magnified). CD8+ T cells were counted manually from three different fields of each mice. Bar graphs show the mean ± SD, representing two independent experiments. ^*^p < 0.05. ^**^p < 0.01. ^***^p < 0.001, ^****^p < 0.0001.

**Figure 4:**
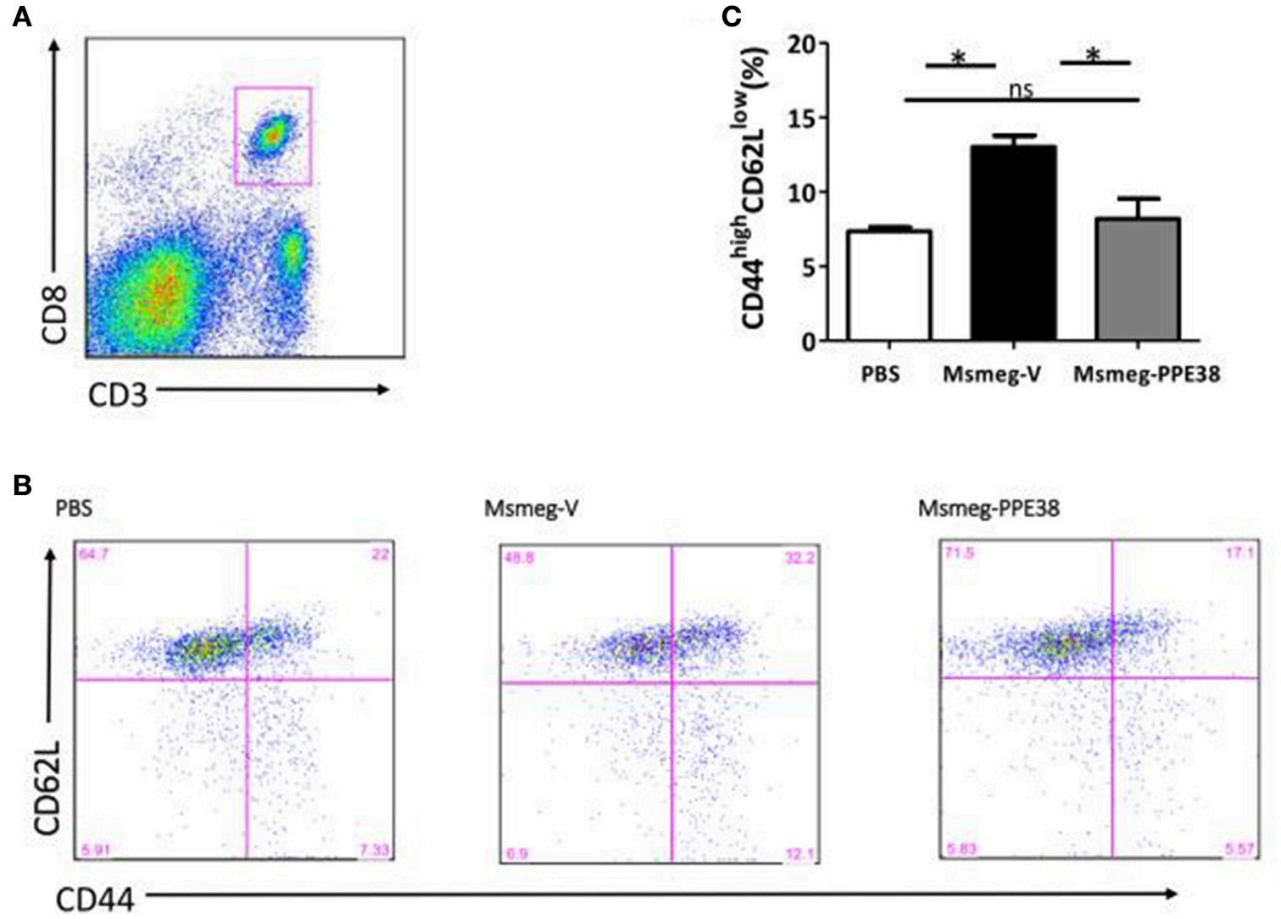
**PPE38 inhibited CD8^**+**^ T response in infected spleen**. Spleen cells derived from three C57BL/6 mice were collected at 6 dpi in each infected group. **(A)** Representative flow cytometry profiles of CD8^+^ T cells which were gated first as CD3^+^/CD8^+^ cells **(B)** he expression of cell surface markers of CD8^+^ T cells including CD44 and CD62L was examined by FACS analysis using the respective PE-Cy7 or PerCP-Cy5.5 mAbs. **(C)** Bar graphs show the mean ± SD percentages of CD44^high^CD62L^low^ CD8^+^ T cells, representing two independent experiments. ^*^p < 0.05.

CD8^+^ T cells will be recruited to infected organs post-MTB infection (Tully et al., 2005). To further characterize the PPE38 protein function in MHC class I restricted CD8+ T cells activation *in vivo*, the distribution of CD8^+^ T cells in different mouse tissues was investigated. At 6 dpi, the number of CD8^+^ T cells in the spleen (Figure 3A), liver (Figure 3B) and lungs (Figure 3C) of mice infected by Msmeg-PPE38 was significantly lower than that in the control strain. Strikingly, we found that spleens isolated from Msmeg-V infected mice were much larger than those obtained from the PBS and Msmeg-PPE38 groups (data not shown). CD44 expression is an indicative marker for effector/memory T-cells. To assess whether decreased MHC class I expression mediated by PPE38 influenced specific stimulation of CD8^+^ T cells from infected mice, we used FACS to analyze the surface expression of CD62L and CD44 on CD8^+^ T cells. Effector/memory T cells were reported previously to exhibit a CD44^high^CD62L^low^ phenotype (Gerberick et al., 1997). The generation of effector/memory T cells was increased, in line with up-regulated CD44 expression in CD8^+^ T cells from Msmeg-V-infected mice, compared with PBS control. This effect was abrogated when PPE38 was expressed (Figure 4). Together, these findings demonstrated that PPE38 inhibited macrophage MHC class I expression and were associated with a decreased number of effector/memory CD8+ T cells *in vivo*.

### Survival of *M. smegmatis* in C57BL/6 mice is enhanced when expressing recombinant PPE38 protein

**Figure 5:**
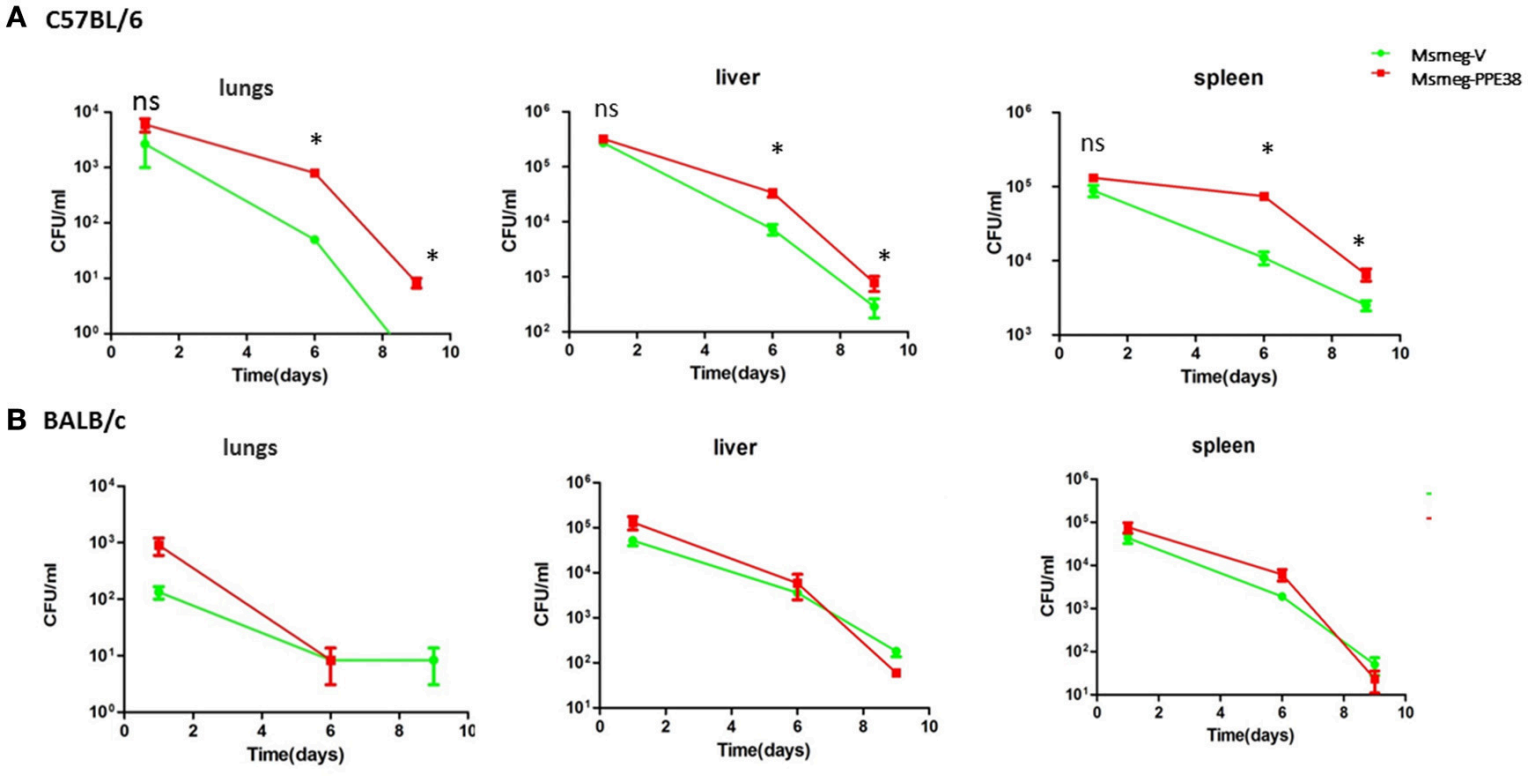
***M. smegmatis*** **survival is enhanced in the C57BL/6 mice by expressing PPE38**. C57BL/6 mice and BALB/c mice were infected with MS-PPE38 and MS-V (2 × 10^7^), and then the bacteria loads in lungs, liver and spleen were counted at 1, 6, and 9 dpi of C57BL/6 mice **(A)** and BALB/c mice **(B)**. Data represent mean ± SD of five mice per group for each time point. ^*^p < 0.05.

We next assessed the effect of PPE38 on the survival of *M. smegmatis* in C57BL/6 mice. Msmeg-V or Msmeg-PPE38 at 2 × 10^7^ colony forming units (CFUs) were injected into the mice intraperitoneally. The initial bacteria load was verified for Msmeg-RvPPE38 and Msmeg-V in the spleen, liver, and lungs at 1 dpi (Figure 5A). Further CFU counts at 6 and 9 dpi indicated that *M. smegmatis* expressing PPE38 persisted for a significantly longer time period than Msmeg-V in all the organs, which is correlated well with the above tissue immunohistochemical data. The CFU of the MS-PPE38 strain was significantly higher than that of the MS-V strain in all three organs at both 6 and 9 dpi. In the lungs of Msmeg-V-infected mice, the mean CFU counts were significantly lower at 6 dpi as compared with those of infected with Msmeg-PPE38 strain and this trend also continued to the later time point (9 dpi). Similar observations were made in the liver and spleen, although the CFUs at 9 dpi were not as significant as those at 6 dpi. Here, we also assessed the survival of Msmeg-V and Msmeg-PPE38 in BALB/c mice (Figure 5B), while no difference was observed. This observation might be due to the limited function of manipulating MHC class I haplotypes by PPE38 on a BALB/c background (Flynn et al., 1995). These data suggested that PPE38 plays a vital role in mycobacterial virulence, through dampening MHC class I response.

## Discussion

In the present study, we attempted to determine the role played by PPE38 in inhibiting mycobacterial Ag presentation by macrophages. Our data showed that the expression of MHC class I in infected macrophages was inhibited by the *Mycobacterium* PPE38 protein, which in turn influenced the numbers of CD8^+^ T cells.

Although macrophages are known as antigen presenting cells (APCs) that could present Ags to CD8^+^ T cells (Bermudez et al., 1997, Teitelbaum et al., 1999; Overbergh et al., 2000), the mechanisms whereby MTB is processed for presentation by MHC class I molecules and how this process is regulated remain unclear. In the current study, as measured by real-time quantitative PCR and FACS, we found that the expression of MHC class I on PMs was inhibited after exposure to *M. smegmatis* expressing PPE38. We provide the evidence that PPE38 can directly suppress MHC class I expression. In addition, PPE38-expressing Msmeg intraperitoneal infection also rapidly induces a population of CD11b^low^MHC classI^low^ subsets which largely explains the significant decrease of MHC class I on PMs in Msmeg-PPE38 group compared to Msmeg-V (Figures 2A,B). Thus, Msmeg-PPE38 could affect MHC class I expression on PMs through several ways *in vivo*. More studies are warranted to dissect the underlying mechanisms for different situations.

The role of PPE38 protein in MTB pathogenesis might be correlated with the genetic background of the host. In general, C57BL/6 mice are relatively more resistant to MTB infection than BALB/c mice (Flynn et al., 1995; Chackerian and Behar, 2003; Arko-Mensah et al., 2009; Sergio et al., 2015). In the current study, we have observed that the MTB PPE38 protein only enhanced the survival of *M. smegmatis* in C57BL/6 mice instead of BALB/c mice. One possible reason for this discrepancy is due to the different MHC class I haplotypes (Flynn et al., 1995). In addition, C57BL/6 mice generate a stronger T helper (Th) 1 immune response, while BALB/c mice favor a Th2-driven immune response (Kramnik et al., 2000; Watanabe et al., 2004). While Th1 cytokines have been implicated for host resistance against MTB (Munk and Emoto, 1995; Hernandez-Pando et al., 1997; Power et al., 1998), certain Th1 inflammatory cytokines could play a deleterious role in anti-mycobacterial immunity under certain conditions. For instance, our previous study indicated that PPE38 promoted the secretion of host Th1 cytokines, especially TNF-alpha, and led to severe pathogenesis in *M. marinum*-infected zebrafish, including both tissue damage and bacterial dissemination (Dong et al., 2012). In agreement with this, we found that the expression of PPE38 in *M. smegmatis* could provide a benefit for the survival of these bacteria in C57BL/6 mice, but not BALB/c mice. More study is needed to further explore this issue.

Tissue-resident macrophages are a heterogeneous population(Sieweke and Allen, 2013). After infection with Msmeg-V and Msmeg-PPE38, C57BL/6 mouse PMs were analyzed by fluorescence activated cell sorting (FACS) marked as CD11b^+^F4/80^+^. As reported before (Ghosn et al., 2010), two PMs subsets defined as CD11b^high^F4/80^high^ and small PMs CD11b^low^F4/80^low^ coexist in infected C57BL/6 mice via intraperitoneal injection (Figure 2). CD11b^high^F4/80^high^ PMs was dominant in PBS group, but disappeared following infection of *M. smegmatis* strains. Interestingly, CD11b^low^F4/80^low^ PMs were generated rapidly in Msmg-PPE38-infected mice as early as 6 hpi. We speculate that similar CD11b^low^F4/80^low^ PMs could be generated in other organs, including lungs, spleen, and liver. These macrophages express low levels of MHC class I molecules, which may contribute to the lower proliferation of CD8^+^ T cells and lower generation of effector/memory CD8^+^ T cells observed in this study. The recruitment of CD8^+^ T cells could also be affected by these PMs. These ideas can be tested and confirmed in our future studies.

The expression of virulence genes by MTB is largely in a time-dependent manner post infection, which reflects the adaption of bacilli to the changes of the surrounding environment (Ramakrishnan et al., 2000; Mehrotra et al., 2014; Calder et al., 2015). PPE38 was highly expressed after 90 dpi in guinea pigs, but not at the early stage (30 dpi) (Kruh et al., 2010). This might be correlated with the recruitment of CD8^+^ T cells, which only increased significantly after day 40 (Grover et al., 2009). In support of this, our results demonstrated that the proliferation of *M. marinum* in macrophages is independent of PPE38 expression (Figure 3A). In addition, there was no significant difference in proliferation of *M. marinum* WT Mm and the PPE38 mutant strains at 2, 4, and 7 dpi in zebrafish larvae, indicating that PPE38 might have only a trivial function in evading host innate immune responses (Figures S3B,C). By contrast, during the infection of adult zebrafish, which have adaptive immunity, the PPE38 mutant was attenuated significantly compared with the WT Mm (Dong et al., 2012), indicating the indispensable role of PPE38 in interfering with host adaptive immune responses.

Until now, the mechanisms of the protective immune response against TB have not been fully understood. Only few MTB proteins were proved to play a role in inhibiting alternate Ag processing. 19kD lipoprotein, plays role in inhibiting alternate MHC class I Ag processing of macrophages, while without disturbing MHC class I expression (Tobian et al., 2003). Recently, PE_PGRS47 was identified as an factor which could inhibit class II-restricted Ag presentation by dendritic cells (Saini et al., 2016). Our study showed that fewer MHC class I was expressed when MTB PPE38 was expressed in *M. smegmatis*. Also, PPE38 participates in manipulating CD8^+^ T cells response, which in turn benefits *M. smegmatis* survival in host. These findings from this study helped us to better understand the unique function of PPE38 protein on host immunity, and provide an ideal target for future tuberculosis vaccine design.

## Ethics statement

This study was carried out in accordance with the recommendations of the Guidelines for the Care and Use of Laboratory Animals from the National Institutes of Health and were approved by the Animal Care and Use Ethical Committee of Fudan University. The protocol was approved by the Animal Care and Use Ethical Committee of Fudan University.

## Author contribution

LM and QG designed the project and wrote the main manuscript text. LM and JT performed Figures 1–4, Figures S1,S2. HW perform Figure 5 and Figures S3A,B. CT did FACS analysis. QW and CN prepared Figures S3C,D and analyzed data. LM and XZ analyzed data. QG and XZ supervised the experiments. All authors reviewed the manuscript.

## Funding

This study was supported by grants from the National Natural Science Foundation of China (No. 81271790 and 31270961), External Cooperation Program (No. GJHZ201312) and Interdisciplinary Innovation Team from Chinese Academy of Sciences.

### Conflict of interest statement

The authors declare that the research was conducted in the absence of any commercial or financial relationships that could be construed as a potential conflict of interest.
