# Supplementary figures and images for: PPE38 Protein of Mycobacterium tuberculosis Inhibits Macrophage MHC Class I Expression and Dampens CD8+ T Cell Responses
Source: Front Cell Infect Microbiol. 2017 Mar 13;7:68. doi: 10.3389/fcimb.2017.00068 (PMC5346565; doi:10.3389/fcimb.2017.00068)

**A**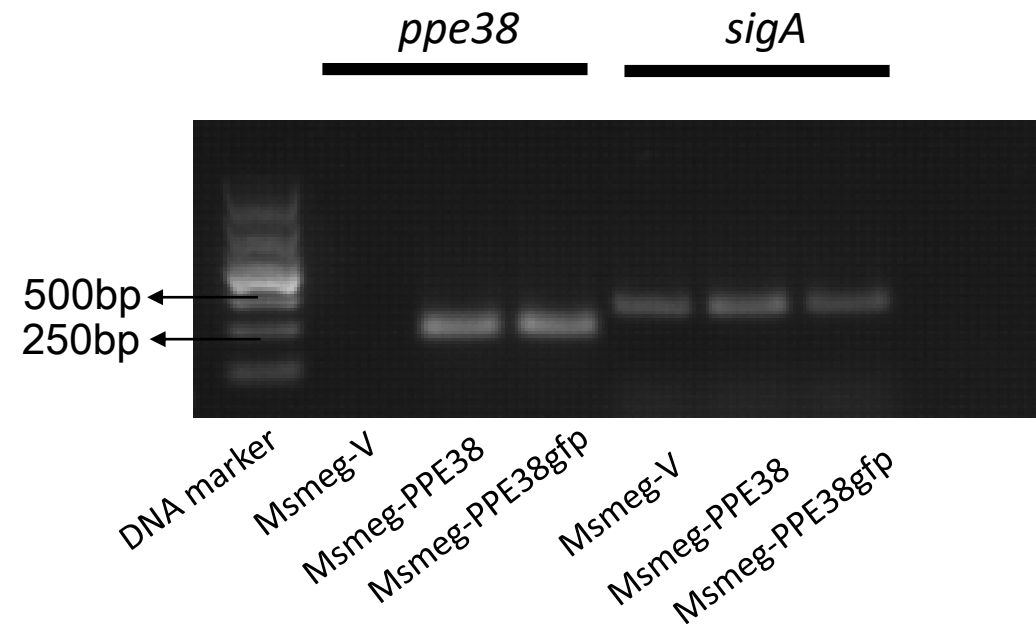**B**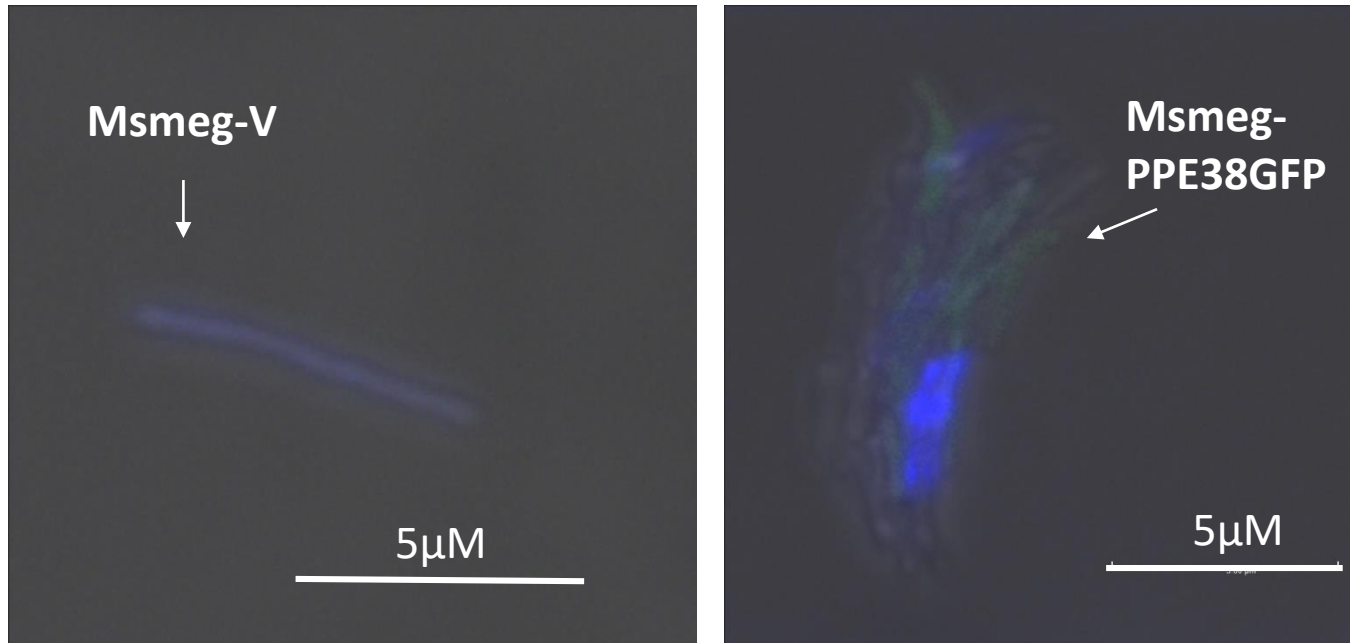

Supplement: Supplementary file 1 [file DataSheet2.PDF]

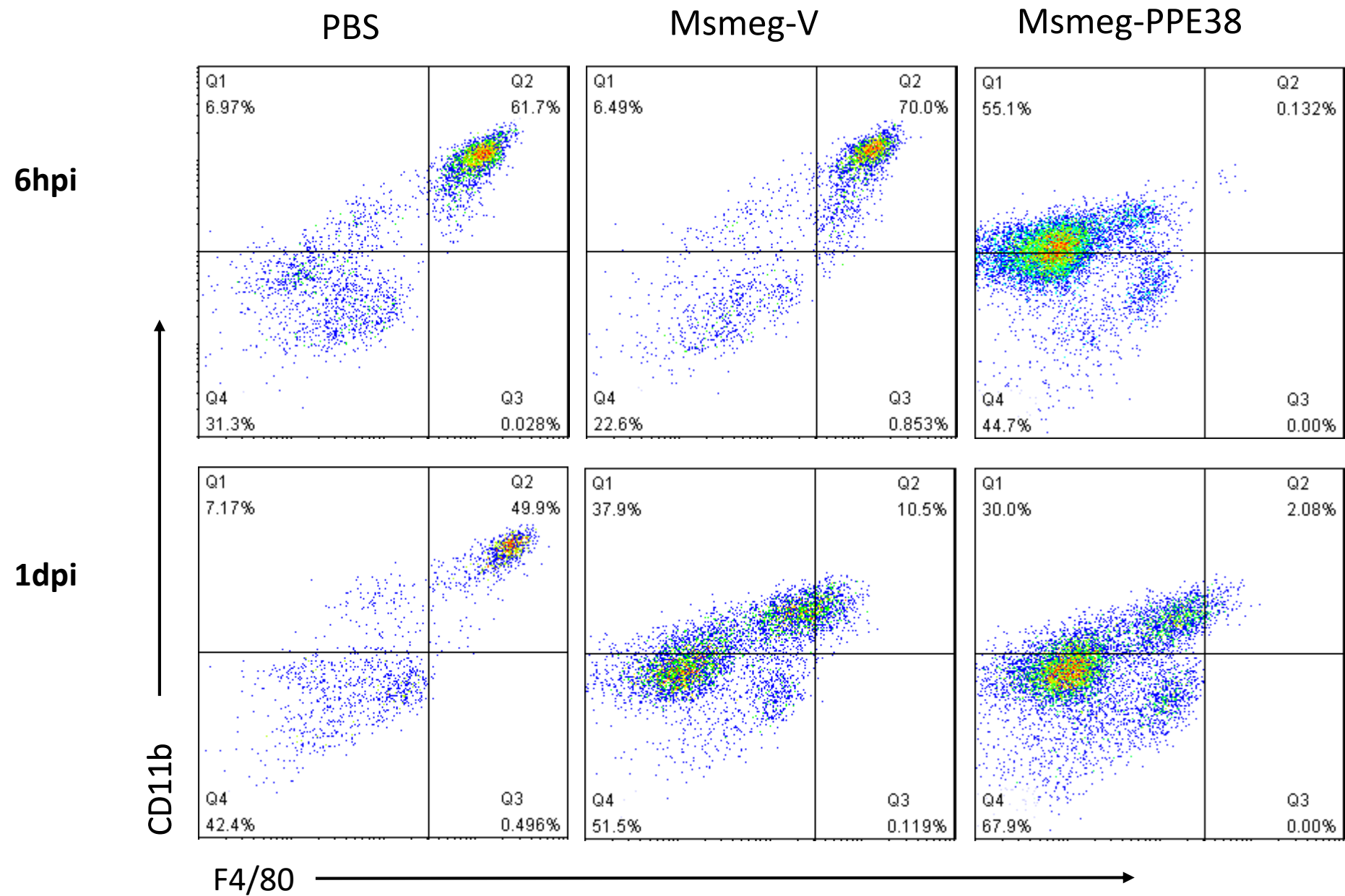

Supplement: Supplementary file 2 [file DataSheet3.PDF]

**A**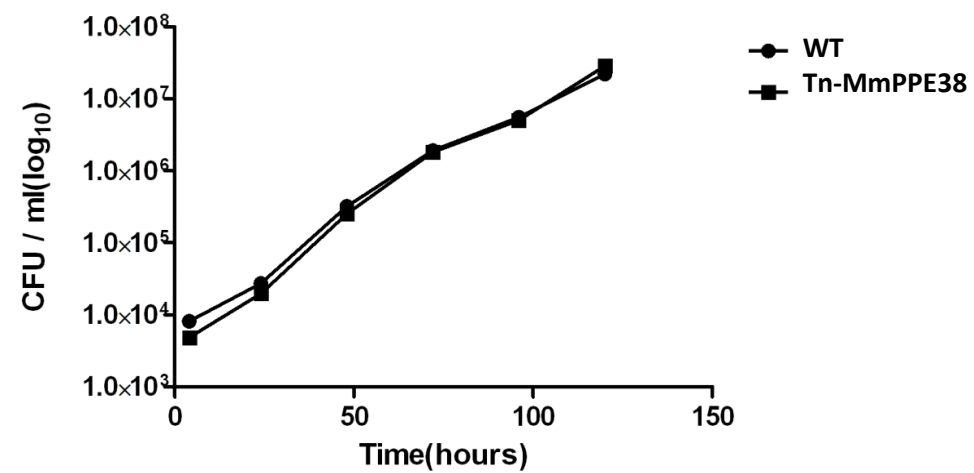**C**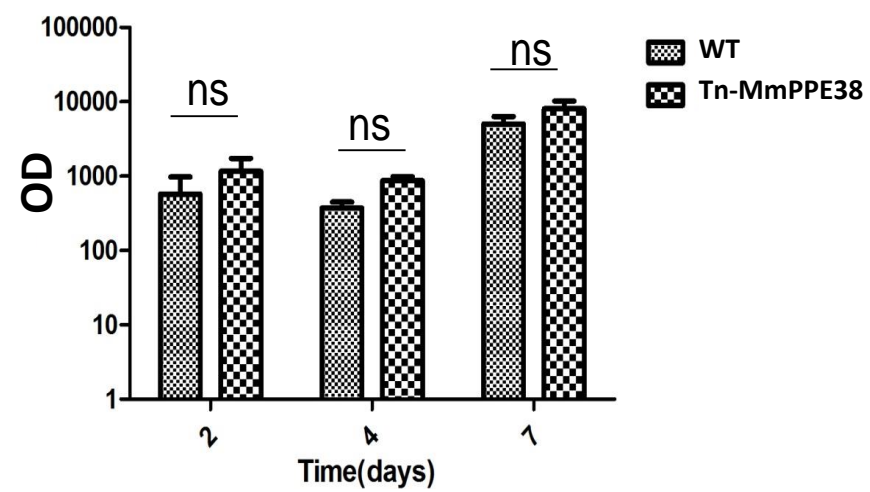**B**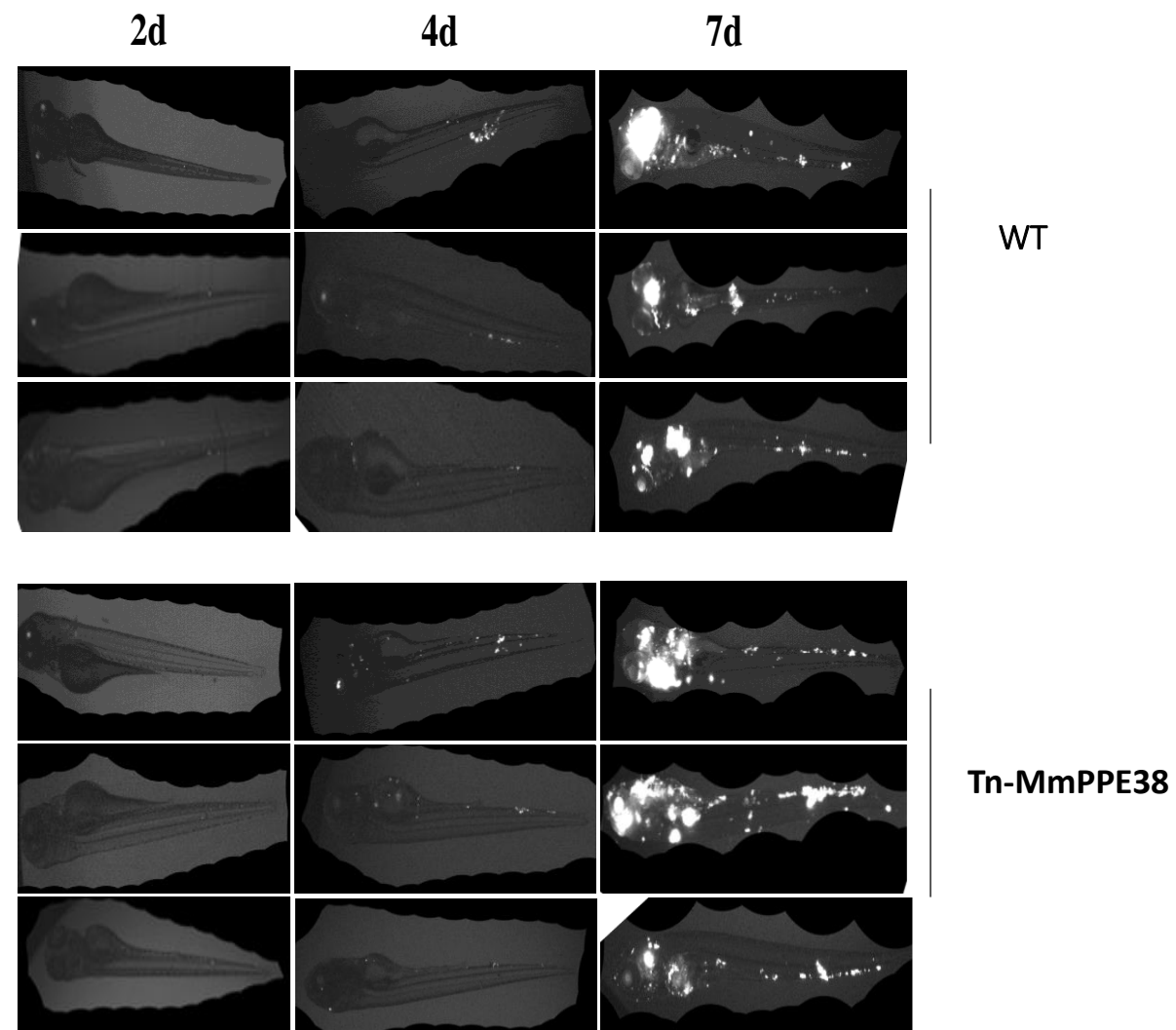

Supplement: Supplementary file 3 [file DataSheet4.PDF]
